# Effects of microRNAs on angiogenesis in diabetic wounds

**DOI:** 10.3389/fmed.2023.1140979

**Published:** 2023-03-20

**Authors:** Bailey D. Lyttle, Alyssa E. Vaughn, James R. Bardill, Anisha Apte, Lauren T. Gallagher, Carlos Zgheib, Kenneth W. Liechty

**Affiliations:** ^1^Laboratory for Fetal and Regenerative Biology, Department of Surgery, School of Medicine, University of Colorado Denver—Anschutz Medical Campus, Aurora, CO, United States; ^2^Laboratory for Fetal and Regenerative Biology, Department of Surgery, College of Medicine, University of Arizona Health Sciences College of Medicine—Tucson, Tucson, AZ, United States

**Keywords:** angiogenesis, diabetes mellitus, microRNA, diabetic foot ulcers, chronic wounds, wound healing

## Abstract

Diabetes mellitus is a morbid condition affecting a growing number of the world population, and approximately one third of diabetic patients are afflicted with diabetic foot ulcers (DFU), which are chronic non-healing wounds that frequently progress to require amputation. The treatments currently used for DFU focus on reducing pressure on the wound, staving off infection, and maintaining a moist environment, but the impaired wound healing that occurs in diabetes is a constant obstacle that must be faced. Aberrant angiogenesis is a major contributor to poor wound healing in diabetes and surgical intervention is often necessary to establish peripheral blood flow necessary for healing wounds. Over recent years, microRNAs (miRNAs) have been implicated in the dysregulation of angiogenesis in multiple pathologies including diabetes. This review explores the pathways of angiogenesis that become dysregulated in diabetes, focusing on miRNAs that have been identified and the mechanisms by which they affect angiogenesis.

## 1. Introduction

Diabetes mellitus is a growing global epidemic with prevalence approaching 10% of the worldwide population (1). Diabetic foot ulcers (DFU) are chronic wounds that afflict nearly a third of diabetic patients within their lifetime (2). The pathogenesis of DFU is multifactorial and major contributors include peripheral neuropathy, external trauma, immunosuppression, edema, and peripheral arterial disease (3–5). DFU are associated with significant morbidity—over 80% of amputations that occur in diabetic patients are preceded by DFU, and as many as 29% of diabetic patients diagnosed with DFU will eventually require amputation (5–8). Mortality of DFU is also high, and some DFU classifications are associated with a 5 years mortality rate of 55% (8). DFU are additionally associated with tremendous costs to healthcare systems at upward of $13 billion annually in the United States alone (9, 10). Treatment of DFU can be challenging in the setting of chronic inflammation, immunosuppression, and poor angiogenesis (11). Currently, the mainstays of treatment include pressure offloading, antimicrobial therapy in the setting of infections, tissue debridement, and maintenance of a moist wound environment (3, 5, 11, 12). Surgical interventions are often necessary, including tendon lengthening procedures, removal of bony prominences, and reconstructions that aim to offload pressure from the ulcerating areas, but these often result in secondary ulceration and progress to require amputation (11). Vascular interventions including angioplasty, stents, and surgical bypass are also frequently needed in this patient population to improve peripheral blood flow to the affected area (5, 11). Pharmacological options for treating DFU are currently limited. Becaplermin (Regranex^®^, Smith & Nephew), a topical formulation of recombinant human platelet-derived growth factor (rhPDGF), is the only FDA-approved topical treatment presently available, but clinical efficacy has been limited (13). In recent years, pathologic angiogenesis associated with diabetic wounds has been identified as a major player and potential target for treatment (14). MicroRNAs (miRNAs), short non-coding RNAs that play a critical role in gene expression, have been implicated in angiogenesis in many disease processes including cancer, arthritis, and ocular disease and have been linked to aberrant angiogenesis in diabetic wounds (15–18). This review explores the many factors that play a significant role in angiogenesis, focusing on those that become deranged in diabetic models, and discusses the mechanisms of miRNAs that have been identified as potential targets for treatment.

## 2. Angiogenesis in wound healing

**FIGURE 1:**
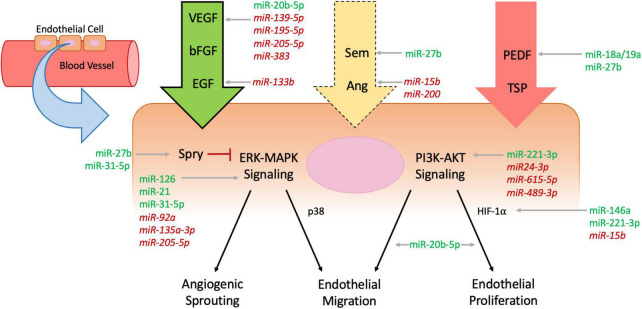
Mechanisms of angiogenesis affected by microRNAs. Endothelial cells line blood vessel walls and are the main cells affected by processes of angiogenesis. Circulating pro-angiogenic factors (green arrow, bold outline), anti-angiogenic factors (red arrow, no outline), and mixed factors (yellow arrow, dashed outline) all interact with endothelial cells and influence intracellular signaling including the extracellular signal-regulated kinases-mitogen activated protein kinases (ERK-MAPK) and phosphatidylinositol 3-kinase (PI3K)-Protein Kinase B (AKT) pathways, which ultimately regulate angiogenic sprouting and endothelial migration and proliferation. Intracellular protein Sprouty (Spry) inhibits ERK-MAPK signaling to downregulate angiogenesis (red flathead arrow). MicroRNAs (MiRNAs) influence angiogenesis throughout the cellular pathways—pro-angiomiRs are denoted in green plain text and anti-angiomiRs in red italicized text.

Angiogenesis is the process by which the body forms new capillary blood vessels. As the human body is dependent on the capillary system for gas exchange as well as the transfer of nutrients and waste between blood vessels and tissue, its development and regeneration are of the utmost importance. While the mechanisms of angiogenesis have been speculated since the days of Leonardo da Vinci, interest in its processes has grown vastly over recent years given the involvement of angiogenesis in many disease pathologies (14, 19, 20). A multitude of biomarkers and pathways that appear vital to the process of angiogenesis have been implicated through the vast amount of research conducted on angiogenesis. Blood vessels exist at baseline in a quiescent state with low circulating levels of proangiogenic factors, such as vascular endothelial growth factor (VEGF) and fibroblast growth factor (FGF), balanced with antiangiogenic factors that help maintain a state of homeostasis regarding growth (14, 21). When an injury to a blood vessel occurs, a state of hypoxia is induced and endothelial cells are disrupted, which together induce an influx of inflammatory cells to the site of the injury (22). An inflammatory milieu is produced with a variety of cytokines and other proangiogenic factors flooding the injured area, and the interactions between these proangiogenic factors, endothelial cells, and extracellular matrix (ECM) proteins are critical to inducing angiogenesis in the wound (21, 23). As wound healing proliferates, granulation tissue is formed and acts as the bed in which angiogenesis can occur (24). Here we highlight the key cell types, as well as both proangiogenic and antiangiogenic factors, and their involvement in the mechanistic pathways by which angiogenesis in wound healing occurs (Figure 1).

### 2.1. Cell types involved in angiogenesis

Endothelial cells form the semipermeable barrier that lines the vascular system and act as secretory cells that release a variety of cytokines involved in hemostasis, inflammation, and maintenance of vascular tone (25, 26). When endothelial cells are disrupted, the release of proangiogenic factors shifts endothelial cells from their quiescent state to one of active proliferation. Endothelial cells are lined with receptor tyrosine kinases including VEGF receptors (VEGFR) and FGF receptors (FGFR), which upon phosphorylation activate several downstream pathways including the extracellular signal-regulated kinases-mitogen activated protein kinases (ERK-MAPK) and phosphatidylinositol 3-kinase (PI3K) pathways to induce endothelial cell proliferation (27).

Pericytes are mural cells that wrap around endothelial cells to form the basement membrane of capillary vasculature (14). They were originally morphologically linked to vascular smooth muscle given the presence of mutual surface markers such as alpha smooth muscle actin (α-SMA) and function similarly to smooth muscle cells within the capillary vessel walls (14, 28). When injury occurs, pericytes are activated by factors such as platelet derived growth factor (PDGF) and transforming growth factor-beta (TGF-β) which induce detachment of pericytes that subsequently migrate into the parenchyma where they act as a guide for vascular sprouting (29). Additionally, the hypoxic environment created following injury induces pericytes to secrete metalloproteinases (MMPs), which release endothelial cells from the basement membrane, allowing for endothelial cell migration into the wounded area (30).

Fibrocytes, originally described in 1994, are circulating fibroblast-like cells of hematopoietic origin that bear surface markers including CD13, CD34, CD45, and α-SMA (14, 31). Fibrocytes have been identified to secrete proangiogenic factors such as VEGF, PDGF, and FGF, and similarly to pericytes, fibrocytes also secrete MMPs that contribute to the degradation of the ECM for mobilization of endothelial cells (32, 33). The detailed mechanistic effects of fibrocytes on angiogenesis are still being elucidated, but animal models have demonstrated fibrocyte-driven improved wound healing and upregulation of proangiogenic factors VEGF and FGF (34). Additionally, treatment of wounded rats with injected FGF induced improved arteriole development and increased formation of capillary structures composed of CD34+ fibrocytes, suggesting that FGF stimulation specifically of CD34+ fibrocytes may be a key portion of angiogenesis in wound healing (35).

### 2.2. Proangiogenic factors

Vascular endothelial growth factor is a glycoprotein similar in structure to PDGF (36). While VEGF has a multitude of functions including the stimulation of endothelial cell division and chemotaxis, its most notable function is its involvement in angiogenesis (37). VEGF is released in large quantities in response to tissue injury and hypoxia, specifically due to the induction of hypoxia inducible factor 1 (HIF-1), which transcribes VEGF *via* the PI3K pathway (21, 36, 38). VEGF subsequently binds to VEGFR1 and VEGFR2 on endothelial cells (39). The resultant phosphorylation of tyrosine kinase residues on the receptors activates the MAPK pathway which induces endothelial proliferation and vessel growth, while p38 activation induces cellular migration (40). VEGF notably upregulates B-cell lymphoma 2 (Bcl-2), an anti-apoptotic protein expressed on endothelial cells that has been associated with improved angiogenesis and enhanced endothelial cell survival (41, 42).

Fibroblast growth factor was first noted to have proangiogenic properties in 1984, predating the identification of VEGF as such by several years (43). The role of FGF in angiogenesis was initially questioned as FGF lacks a transmembrane sequence and subsequently cannot be secreted, though alternative modes for cellular release have been proposed as well as mechanisms of release *via* cellular injury (21, 44). Similarly to VEGF, FGF enacts its effects by binding to receptor tyrosine kinases and activating numerous factors such as p38, PI3K, and MAPK with resultant upregulation of downstream transcription and stimulation of endothelial cell migration and proliferation (45).

Epidermal growth factor (EGF) is a 53-amino acid protein and was one of the earliest identified growth factors, originally derived from the salivary glands of mice in 1962 (46, 47). EGF acts on the EGF receptor (EGFR), and its major function in wound healing is stimulation of epidermal cell regeneration and keratinocyte proliferation and migration (48). Similarly to VEGF and FGF, EGF stimulates the ERK-MAPK and PI3K-Protein Kinase B (AKT) signaling pathways intracellularly to produce its effects (46). Regarding its role in angiogenesis, EGF was found to induce neovascularization in a rabbit cornea model in 1979 (49). EGF has specifically been implicated in endothelial cell maturation and pericyte migration and has also been shown to directly induce VEGFA production (50, 51).

### 2.3. Antiangiogenic factors

Pigment epithelium derived factor (PEDF) is an endogenous secreted antiangiogenic glycoprotein that is predominantly produced by fibroblasts (52). PEDF mainly elicits its antiangiogenic effects by targeting and inhibiting endothelial cell proliferation while promoting apoptosis (53). Wounds in PEDF^–/–^ mice have significantly increased development of capillaries and delayed capillary pruning, and *in vitro* studies of PEDF demonstrate decreased expression of surface receptors associated with angiogenesis, suggesting that PEDF acts through multiple mechanisms to inhibit vascular growth (54).

Thrombospondins (TSP) are glycoproteins that act to downregulate angiogenesis. Originally discovered as a protein released from platelets in response to thrombin stimulation, TSP has been the target of antiangiogenic research and identified to inhibit angiogenesis through multiple mechanisms (55). TSP was initially associated with inhibition of angiogenesis through its inhibition of endothelial proliferation and migration (56). TSP inhibits endothelial cell proliferation by binding to FGF and VEGF, preventing their interaction with the ECM and mitigating their mitogenic effects (57). Apoptosis of endothelial cells is induced by TSP activating CD36 which induces transcription of caspases, and when bound to very low density lipoprotein receptor (VLDLR), TSP interacts directly with VEGF bound to its receptor to inhibit the MAPK and PI3K pathways and halt endothelial cell cycle progression (58).

Sprouty (Spry1-4) is a family of intracellular proteins that provide negative feedback modulation of the ERK-MAPK cellular pathways (59). Spry1, Spry2, and Spry4 have all been identified to repress VEGF- and FGF-mediated MAPK activation, thereby suppressing endothelial cell migration and proliferation (60, 61). Expression of Spry1 has been shown to increase significantly in cell cultures exposed to hypoxia and results in upregulation of cell cycle inhibitors p21 and p27 (62). Additionally, mouse wounds treated with a topical gel formulation containing Spry2 exhibited impaired angiogenesis with inhibited endothelial cell migration and decreased MAPK signaling (59).

### 2.4. Mixed factors

Semaphorins (Sem1-8) are a family of membrane-bound and secreted proteins with multiple classes that bind to plexins and neuropilins to regulate neural development and immune response in addition to angiogenesis (63). Sem3 proteins have been implicated in inhibition of angiogenesis through regulation of endothelial cell survival and migration (64). Sem6 proteins have alternatively been identified to promote angiogenesis through the enhancement of both VEGF and FGF signaling (65, 66). However, Sem6 proteins have also been associated with inhibition of endothelial cell growth and angiogenesis in tumors, though this mechanism remains undefined (63). Sem6A has specifically been shown to be expressed on endothelial cells and promote angiogenesis *via* VEGFR signaling, and silencing of Sem6a in both culture and animal models results in upregulated endothelial cell death (63). The mixed role of Sem6a demonstrates that its impact on angiogenesis requires further elucidation.

Angiopoietins (Ang 1-4) are a family of glycoproteins present on vascular endothelial cells that regulate angiogenesis. Angiopoietins bind to the Tie-2 tyrosine kinase receptor, a receptor that has been identified as crucial for embryonic vasculogenesis (67). Ang-1 has been implicated in vessel growth and maturation, with transgenic expression of Ang-1 in vessels resulting in increased numbers of endothelial cells as well as pericytes, improved vessel differentiation, and decreased permeability compared to expression of VEGF alone in animal models (68). Ang-2 appears to be key for vascular remodeling as it plays a role in vascular destabilization (69). Ang-2 has been characterized as an antagonist to Ang-1 and Tie-2 receptors, and overexpression in a murine embryo model results in significant disruption of vascular formation (70). However, Ang-2 is also upregulated at sites of angiogenesis in addition to upregulation of VEGF, suggesting some role in neovascularization (69).

## 3. Dysregulation of angiogenesis in diabetes

Disruption of normal angiogenesis is a key feature of numerous pathologic morbidities associated with diabetes mellitus. This disruption in both the macro- and microvasculature stems largely from a state of prolonged hyperglycemia that is characteristic of both undiagnosed and poorly controlled diabetes, and results in upregulated endothelial apoptosis (71–73). Hyperglycemia also induces the formation of advanced glycation end products (AGEs) which, particularly when coupled with hypoxia and oxidative stress, have been associated with vascular disease in diabetes (73).

Notably, dysregulated angiogenesis in diabetes results in both overactive and depressed vascular formation depending on the specific tissue involved. For example, upregulated angiogenesis, specifically *via* upregulation of VEGF, is pathogenically responsible for two of the major complications associated with diabetes: diabetic retinopathy and diabetic neuropathy (74). Diabetic retinopathy is a progressive disorder characterized by microaneurysms, hemorrhage, and edema that can ultimately result in blindness (74). The loss of retinal capillaries secondary to pericyte destruction results in chronic hypoxia, which induces aberrant angiogenesis that produces fragile vessels which are prone to further rupture and propagation of the same cycle (74–76). Diabetic nephropathy is currently the leading cause of kidney disease necessitating renal replacement therapy and affects up to 40% of patients with diabetes (77). Initially, diabetic nephropathy results in a paradoxical increase in the glomerular filtration rate (GFR), which eventually exhausts the renal tissue and progresses to a steady decline in GFR (74). This hyperfiltration phase occurs in the setting of an increased number of glomerular capillaries, as demonstrated in rat studies compared to non-diabetic controls, suggesting that dysregulated angiogenesis plays a role in the pathogenesis of diabetic nephropathy (78). In both diabetic retinopathy and nephropathy, upregulation of VEGF has been implicated. Hypoxia and hyperglycemia, in addition to numerous cytokines upregulated in the diabetic milieu such as TGF-β and interleukin-1 (IL-1), have been shown to induce upregulation of VEGF (79). In diabetic retinopathy, hypoxia-induced HIF1-a stimulates VEGF upregulation, which binds to endothelial surface receptors Flt-1 and Flk-1 with subsequent protein kinase C (PKC) activation, culminating in endothelial cell proliferation and migration (74, 76). Multiple mechanisms have been proposed linking VEGF upregulation to diabetic nephropathy, including VEGF secretion from podocytes that subsequently binds to the glomerular capillary endothelium, as well as PKC-induced stimulation (80, 81). Upregulation of FGF has also been implicated as a contributor to the aberrant angiogenesis found in diabetic morbidities, and FGF levels in diabetic retinopathy have been shown to be high enough to induce endothelial cell stimulation *in vitro* (74, 82).

The most significant morbidity in diabetes related to downregulated angiogenesis is impaired wound healing. Diabetic patients are prone to wound formation, particularly in the setting of diabetic neuropathy where patients may be insensate to traumatic stimuli, and the wound fails to progress to the later stages of healing (4, 74). While multiple mechanisms have been described as contributory to impaired wound healing in diabetes, one of the major mechanisms implicated is downregulated angiogenesis (6). Impaired angiogenesis in diabetes can occur at any point during the many necessary steps of neovascularization, and numerous cytokines involved in angiogenesis have been described as altered in diabetes (74). VEGF mRNA expression and protein levels are reported to be markedly lower in murine models of diabetes compared to controls (83, 84). Galiano et al. (85) treated diabetic mice with VEGF, which resulted in upregulation of epithelial cell proliferation and ECM deposition as well as increased PDGF-B and FGF mRNA expression levels, though the VEGF-treated wounds were notably leaky and malformed with increased formation of granulation tissue. Macrophages, which produce VEGF, have been found to be dysfunctional with altered phenotypes in diabetic wounds (86). In addition to increased expression of inflammatory cytokines, the pro-inflammatory phenotype of macrophages found in diabetic wounds is also associated with decreased VEGF expression (87). FGF has also been found to be decreased in diabetic wounds resulting in delayed wound healing, suggesting that the diabetic phenotype prevents production of the vital proangiogenic factors that are essential to completion of neovascularization (88). In addition to VEGF and FGF, EGF has been shown to be downregulated in diabetic wounds and has previously been employed as a topical treatment for chronic diabetic wounds (48, 89). PDGF is another proangiogenic factor that has been shown to be dysregulated in diabetes. Both PDGF and PDGF receptor levels are lower in murine models of diabetic wounds (90). Topical administration of PDGF in diabetic wounds has been demonstrated to improve wound healing, and recombinant human PDGF is the basis for the only current FDA-approved topical pharmaceutical treatment for DFU, becaplermin (91, 92). The clinical efficacy of becaplermin has been mixed overall and did have a black box warning for an increased risk of malignancy with repeated use added to its label in 2008, though this warning was notably removed 10 years later after post-market matched cohort studies failed to reproduce the same risk (13, 93, 94).

In addition to downregulation of proangiogenic factors, patients with diabetes also experience upregulation of antiangiogenic factors which further inhibit angiogenesis and wound healing. Qi et al. (95) demonstrated elevated levels of PEDF in both human and murine diabetic wounds, and neutralization of PEDF in a murine model resulted in improved wound healing. Studies have also shown thrombospondins (TSP) to be elevated in human and murine diabetic wounds, and this appears to occur secondary to increased oxidative stress and DNA hypomethylation induced by a high glucose environment, as demonstrated in cultures of human and rat keratinocytes (96, 97). Angiopoietins (Ang1-4), which modulate angiogenesis through both upregulation and downregulation of factors such as VEGF depending on the subtype, contribute to the complex dysregulation of diabetic wound healing as well. The ratio of Ang-2 to Ang-1 becomes increased in diabetes, shifting the influence of angiopoietins on angiogenesis from a pro-angiogenic to an anti-angiogenic state (98). Ang-2 has been found to be elevated in mouse models of diabetic wounds and continues to increase beyond Day 7, whereas Ang-2 levels notably decrease from Day 7 on in non-diabetic mice (99). Tie-2 receptor expression is also absent in diabetic wounds, suggesting complete dysregulation of the Ang/Tie-2 pathway (99). Furthermore, Balaji et al. (100) demonstrated that treatment of diabetic mice with Ang-1 resulted in improved wound healing with increased epithelialization and neovascularization, suggesting that shifting the angiopoietin ratio back toward a predominantly Ang-1 state during wound healing is key for sufficient angiogenesis.

## 4. Dysregulated microRNAs in diabetic angiogenesis

MicroRNAs (miRNAs) are small, non-coding RNAs of which the majority function to downregulate gene expression by base-pairing with the 3’ untranslated regions (3-UTR) of their target messenger RNAs (mRNAs), thereby inhibiting transcription or inducing mRNA destabilization (101). MiRNAs have been extensively linked to angiogenesis in both *in utero* vascular development and post-ischemic neovascularization (15). The role that miRNAs play in the regulation of angiogenesis stems from either targeting and downregulating expression of proangiogenic factors, thereby impairing angiogenesis (anti-angiomiRs), or by targeting and downregulating expression of antiangiogenic factors, thereby inducing angiogenesis (pro-angiomiRs) (102). Since miRNAs have been implicated in the pathologic development of many disease processes such as tumor progression and atherosclerosis, the same interest has been applied to miRNA involvement in the pathogenesis of diabetic morbidities (15–18). Over recent years, numerous pro-angiomiRs and anti-angiomiRs with specific involvement in diabetic wound healing have been implicated, which are summarized in Table 1 and detailed below.

**TABLE 1:** Pro-angiomiRs and anti-angiomiRs implicated in pathologic angiogenesis in diabetes mellitus, their identified targets, downstream functions, and the diabetic animal or human models in which potential targets and functions have been identified.

AngiomiR	Identified target(s)	Function(s)	Model(s)	References
**Pro-angiomiRs**				
miR-18a/19a	TSP-1	Inhibition of endothelial apoptosis resulting in upregulation of endothelial proliferation	Human	103–105
miR-21	TIMP	Activation of MAPK/ERK signaling, resulting in upregulation of VEGF, MMPs, and activated myofibroblasts	Rat	106–108, 111
miR-27b	Sem6a, Spry2, TSP-1	Upregulation of VEGF activation of MAPK pathway, resulting in improved endothelial propulsion and angiogenic sprouting	Mouse	113, 114
miR-31-5p	Spry4, SPRED1, SPRED2	Upregulation of keratinocyte migration and proliferation, improved endothelial cell proliferation, downregulation of HIF1AN	Human Mouse Rat	115–119
miR-126	SPRED1	Increased expression of VEGF and p-ERK resulting in fibroblast and endothelial cell proliferation, accelerated epithelialization, activated angiogenesis, and promotion of collagen maturity	Rat	120–122
miR-146a	NFκB	Downregulation of inflammatory markers from NFκB pathway including TRAF6 and IRAK1; upregulation of VEGF, Bcl-2, and HIF-1α; increased endothelial cell proliferation	Mouse	123–125
miR-221-3p	HIPK2	Upregulation of AKT/eNOS signaling, suppression of HIPK2 with subsequent upregulation of HIF1α signaling	Mouse Rat	127–129
**Anti-angiomiRs**				
miR-15b	Bcl-2, HIF1-α, VEGFA	Decreased expression of VEGF, Bcl-2, and HIF-1α that corrects with mesenchymal stem cell treatment, downregulation of Ang-1 and TEK	Mouse	130–135
miR-20b-5p	wnt9	Impaired endothelial migration and proliferation *via* inhibition of β-catenin pathway, downregulation of VEGF transcription	Mouse Rat	137, 138
miR-23	SDF-1	Downregulation of SDF-1; upregulation of iNOS with associated impairment of VEGF	Human Rat	139–141
miR-24-3p	PI3KR3	Downregulation of PI3K/AKT signaling with impaired tube formation and endothelial migration	Human Mouse	142, 143
miR-26a	SMAD1	Reduction of VEGF-induced nitric oxide production, impairment of VEGFR2 signaling, increased cell cycle inhibition *via* p27	Mouse	144–148
miR-92a	ITGA5	Downregulation of capillary tube formation *via* MAPK pathway inhibition	Mouse Pig	149–153
miR-133b	EGFR	Downregulates EGFR signaling with impaired endothelial proliferation and migration	Mouse	155, 156
miR-135a-3p	FAK, HIP1	Inhibition of VEGF activation *via* MAPK pathway blockade and impaired p38 signaling	Mouse	154, 157, 158
miR-139-5p	c-jun, c-fos, HOXA9	Downregulation of proangiogenic factors *via* c-jun/VEGF/PDGF and HOXA9/c-fos pathways, impaired endothelial migration and proliferation	Human Mouse Rat	160–162
miR-195-5p	VEGFA	Reduced endothelial proliferation, direct suppression of VEGF and its downstream effects	Human Rat	163–165
miR-200	GATA2, VEGFR2	Downregulation of Ang-1 and TEK, associated with deficiency of IRE1α	Mouse	135, 166, 167
miR-205-5p	VEGFA	Suppression of VEGF and FGF levels *via* downregulation of ERK signaling, inhibition of cell cycle progression with increased apoptosis	Human Mouse	165, 168–170
miR-383	VEGFA	Impairs endothelial proliferation and migration, inhibits transcription of VEGFA	Mouse	171–173
miR-489-3p	Sirt1	Inhibits PI3K/AKT/eNOS signaling	Rat	174
miR-615-5p	Tie2, IGF-2, MEF2A, RASSF2	Impairment of endothelial cell proliferation and migration, inhibition of VEGF and eNOS signaling	Mouse	175–177

### 4.1. Pro-angiomiRs

Pro-angiomiRs target and downregulate signals and factors that would normally inhibit angiogenesis, thereby impairing their abilities to block angiogenesis resulting in increased capillary formation. In general, some of the major pro-angiomiRs that have been identified include miR-126, the miR-17-92 cluster, miR-130, miR-210, miR-378, and miR-296, which all ultimately upregulate VEGF expression, endothelial cell proliferation and migration, or sprout formation (102). Several of these have been explored in diabetic models, while additional pro-angiomiRs have also been identified in diabetic wound healing.

#### 4.1.1. miR-18a/19a

The miR-17/92 cluster is a group of six miRNAs (miR-17, miR-18a, miR-19a, miR-20a, miR-19b-1, and miR-92a) that are all located on the same non-protein-coding gene *MIR17HG* and conserved across vertebrates (103). The cluster was originally discovered after amplification of the region was identified in b-cell lymphoma, but since then it has been linked to the pathology of numerous other diseases including multiple cancers, multiple sclerosis, and cardiovascular disease (103). Recently, the miR-17/92 cluster has been linked to angiogenesis. Chamorro-jorganes et al. (104) demonstrated that VEGF induces upregulation of the miR-17/92 cluster *via* ERK and Elk-1 activation, with downstream upregulation of endothelial cell proliferation and angiogenic sprouting. Given this association of the miR-17/92 cluster with angiogenesis, Wang et al. (105) explored if any portion of the cluster is upregulated following maggot debridement therapy in diabetic wounds. In both human tissue samples that underwent maggot debridement therapy and human umbilical vein endothelial cells (HUVECs) that were treated with maggot excretions/secretions, transcription of both miR-18a and miR-19a were found to be significantly upregulated (105). Furthermore, treated tissue and HUVECs also had decreased expression of TSP-1, suggesting that miR-18a and miR-19a may reduce thrombospondin expression, thereby inhibiting the effect of TSP on endothelial apoptosis and allow for improved angiogenesis *via* upregulated endothelial cell proliferation (105).

#### 4.1.2. miR-21

MiR-21 has been extensively studied in cancer and has been found to impact apoptosis, cell migration, and invasion in multiple types of tumors (106). In gliomas, miR-21 was shown to regulate *TIMP3*, which inhibits MMPs and has been linked to angiogenesis (107, 108). In wound healing, miR-21 was found to be elevated in healing wounds and to induce keratinocyte migration, an effect that could be reduced *via* endogenous miR-21 knockdown (109, 110). Additionally, miR-21 expression was upregulated in cell cultures exposed to TGF-β, suggesting a multifactorial but overall beneficial role for miR-21 in wound healing (110). Li et al. (111) found that delivery of miR-21 *via* microvesicles significantly improved wound healing in diabetic rats *via* downregulation of *TIMP3* and activation of the intracellular MAPK/ERK signaling pathway. Proangiogenic VEGF was upregulated in animals treated with miR-21, as were MMPs and markers of activated myofibroblasts, α-SMA and n-cadherin, which are found in healing wounds during angiogenesis (111). While angiogenesis appears to be just one of the cellular processes affected by miR-21, there is evidence that this miRNA is considerably linked to appropriate wound healing.

#### 4.1.3. miR-27b

MiR-27b is expressed on endothelial cells, along with its family member miR-27a, and both miR-27a/b have been associated with endothelial sprouting in embryonic vasculogenesis as demonstrated in zebrafish models (112). Mir-27 has been described to enact its proangiogenic effects by downregulating Sem6A which was associated with endothelial cell repulsion and angiogenic sprouting, though as discussed previously Sem6A has been found to have both proangiogenic and antiangiogenic roles depending on the tissue studied (112, 113). In a murine model of retinal neovascularization, miR-27 was studied as a portion of a miRNA cluster also containing miR-23 and miR-24, and it was found that miR-27 also directly targeted antiangiogenic Sprouty2 (Spry2) (113). Mechanistically, it appears that miR-27 works intracellularly to inhibit both Sem6A and Spry2, thereby upregulating VEGFR activation of the MAPK pathway resulting in angiogenesis, as knockdown of miR-27 resulted in the opposite effect (113). Wang et al. (114) analyzed bone marrow derived angiogenic cells (BMACs) from diabetic and non-diabetic mice and found significantly lower levels of miR-27b in the diabetic mice, with improvement in BMAC function as well as wound healing in diabetic mice with the delivery of a miR-27b mimic. Conversely, when miR-27b was inhibited in non-diabetic BMACs, angiogenesis was downregulated (114). Sem6A was elevated in diabetic BMACs and could be suppressed with the administration of the miR-27b mimic, suggesting that the suppression of Sem6A *via* downregulation by miR-27b may be partially responsible for the proangiogenic effects instigated by miR-27b (114). Interestingly, miR-27b was also found to downregulate antiangiogenic TSP-1, implying yet another mechanism through which miR-27b enacts its proangiogenic effects (114).

#### 4.1.4. miR-31-5p

MiR-31 has previously been linked to wound healing through its expression in keratinocytes and moderation of keratinocyte proliferation and migration (115, 116). MiR-31 enacts its effects on keratinocytes by downregulating Spry4 as well as Spred1 and Spred2, thereby upregulating the MAPK pathway intracellularly (116). Expression levels of miR-31are reduced in wounds of diabetic rats as well as the serum of patients with diabetic retinopathy (117, 118). Yan et al. (119) demonstrated reduced expression levels of miR-31-5p in wounds of diabetic mice and subsequently utilized novel milk-derived exosomes as a delivery system for a miR-31-5p mimic. Delivery of miR-31-5p *in vitro* resulted in improved endothelial cell proliferation and accelerated wound healing in diabetic mice (119). Additionally, administration of miR-31-5p was associated with downregulation of HIF1AN, an inhibitor of proangiogenic factor HIF-1, suggesting that miR-31-5p at least partially enacts its wound healing benefits through the promotion of neovascularization (119).

#### 4.1.5. miR-126

MiR-126 has been previously identified as critical to vascular formation in zebrafish through the regulation of the VEGF response in endothelial cells, specifically through the repression of sprouty-related protein (*SPRED1*) and PI3K pathways (120). In diabetic rats treated with negative pressure wound therapy, Zhang et al. (121) found miR-126 to be significantly upregulated with subsequent repressed expression of *SPRED1* and increased expression of VEGF mRNA and protein as well as increased expression of p-eRK, part of the MAPK pathway that regulates endothelial cell migration. Given the association of miR-126 with improved angiogenesis through VEGF stimulation and improved endothelial cell chemotaxis, Tao et al. (122) explored the use of gene expression technology to overexpress miR-126-3p in synovium mesenchymal stem cells (SMSCs), which also have the ability to promote fibroblast proliferation, and used these cells to develop controlled-release exosomes (SMSC-126-Exos). When used *in vitro*, SMSC-126-Exos stimulated proliferation of both fibroblasts and human microvascular endothelial cells (HMEC-1) (122). Additionally, when used in a diabetic rat model, animals treated with SMSC-126-Exos exhibited increased blood vessel formation as well as greater numbers of cells staining for CD31 (a marker of endothelial cells) and α-SMA (a marker of vascular smooth muscle cells), all of which suggests improved angiogenesis in the setting of topical administration of miR-126 (122).

#### 4.1.6. miR-146a

Decreased expression of miR-146a has been implicated in the pathogenesis of diabetic wounds as it has been associated with increased levels of inflammation (123). Knockout of miR-146a results in significantly delayed wound healing, and the mechanism by which miR-146a functions in its anti-inflammatory role has been linked to the NFκB pathway (124). Dewberry et al. (125) explored the use of miR-146a conjugated to free radical-scavenging cerium oxide nanoparticles (CNP-miR146a) in the treatment of diabetic wounds in mice. In addition to the anti-inflammatory effects demonstrated *via* the downregulation of NFκB and its downstream effectors, CNP-miR146a treatment also resulted in significant upregulation of angiogenesis, as demonstrated through increased levels of VEGF and endothelial proliferation (125). Treatment with CNP-miR146a was also associated with increased expression of HIF-1α which may be responsible for the elevated transcription of VEGF, as well as elevated levels of Bcl-2, which may contribute to endothelial cell survival (125).

#### 4.1.7. miR-221-3p

While miR-221/miR-222 has been previously implicated as a potential inhibitor of angiogenesis due to downregulation of endothelial proliferation and migration (102, 126), miR-221-3p specifically has been implicated as a potential pro-angiomiR in diabetic models. Yu et al. (127) used exosomes isolated from bone marrow mesenchymal stem cells (BMSC), some pretreated with atorvastatin, to treat wounds in a diabetic rat model. The rats treated with exosomes from BMSCs that were pretreated with atorvastatin displayed improved angiogenesis, as demonstrated by increased endothelial proliferation and migration, improved tube formation, and upregulation of proangiogenic factors including PDGF, EGF, bFGF, and Ang-1 both *in vivo* and *in vitro* (127). When further characterizing the mechanism by which the atorvastatin-pretreated BMSCs induced improved angiogenesis, Yu et al. (127) found that these cells activated the AKT/eNOS signaling pathway *via* upregulation of miR-221-3p, and inhibition of miR-221-3p in an *in vitro* model reversed the pro-angiogeneic effects that had been previously demonstrated. Using a similar method, Xu et al. (128) isolated exosomes from endothelial progenitor cells (EPCs) and found high expression of miR-221-3p, these EPC-derived exosomes were subsequently used in the treatment of murine diabetic wounds. Wound healing in both diabetic mice and controls was significantly improved with EPC-derived exosome treatment as well as treatment with miR-221-3p alone, and wounds demonstrated increased expression of VEGF, CD31, and Ki67 (a marker of cell proliferation), suggesting that improved angiogenesis secondary to upregulation of miR-221-3p was responsible for more rapid wound closure (128). In a subsequent study, Yu et al. (129) found that miR-221-3p promoted angiogenesis in HUVECs through downregulation of homeodomain-interacting protein kinase 2 (HIPK2), which downregulates angiogenesis by mediating the HIF-1α signaling pathway and is overexpressed in HUVECs cultured in high glucose to mimic a diabetic environment. When murine diabetic wounds were treated with a miR-221-3p agonist, HIPK2 expression was downregulated in wound margin tissue and overall wound healing was improved (129).

### 4.2. Anti-angiomiRs

In contrast to pro-angiomiRs, anti-angiomiRs target and downregulate factors and cytokines that promote angiogenesis, thereby downregulating the formation of neovasculature. Some miRNAs that have been previously identified as anti-angiogenic include miR-15b, miR-16, miR-20, miR-24, miR192, miR195, miR-221/miR-222, and miR-328, which either target VEGF directly, impair endothelial cell migration, or induce endothelial cell apoptosis, though the majority of these studies occurred in models of cancer (102). As with pro-angiomiRs, some of these miRNAs have also been identified as anti-angiogenic specifically in diabetic models, and several new anti-angiomiRs have been described over recent years.

#### 4.2.1. miR-15b

MiR15-b, along with miR-16 and miR-20, was initially identified *via* computational analysis as a potential inhibitor of VEGF by analyzing target regions in the 3’-UTR of VEGF and then analyzing matching miRNA binding sites (130). Once these miRNAs were identified, their impact on VEGF was validated on a cellular model using a cell line derived from nasopharyngeal carcinoma, and transfection with miR-15b, miR-16, and miR-20 mimics was associated with 25–50% reduced levels of VEGF, while inhibition of endogenous miR-15b, miR16, and miR-20 induced the opposite effect (130). MiR-15b has been found to impair tumor angiogenesis *via* inhibition of VEGF, and low circulating levels of miR-15b were associated with increased levels of VEGF in patients with diabetic retinopathy compared to controls (131, 132). Xu et al. (133) analyzed miR-15b levels in diabetic mouse wounds and found them to be significantly elevated and associated with decreased levels of Bcl-2, VEGF, and HIF-1α. Additionally, treatment of the diabetic wounds with mesenchymal stem cells resulted in reversal of these effects with downregulation of miR-15b and subsequent upregulation of Bcl-2, VEGF, and HIF-1α (133). In a more recent study, Xu et al. (134) describe an additional mechanism through which miR-15b regulates angiogenesis, specifically by targeting and downregulating stromal cell-derived factor 1α (SDF-1α), which is a CXC chemokine that is upregulated by HIF-1α and acts on CXC receptor type 4 (CXCR4) to recruit hematopoietic stem cells to sites of injury. After identifying SDF-1α downregulation in diabetic wounds, Xu et al. (134) used a CXCR4 agonist to treat diabetic human dermal fibroblasts and found reduced overexpression of miR15b, and subsequent treatment of murine diabetic wounds with the same CXCR4 agonist demonstrated a significantly improved rate of wound closure compared to untreated controls. Together, these studies identify miR-15b as a potential anti-angiogenic treatment target for diabetic wounds. Pizzino et al. (135) utilized anti-miR-15b, either alone or in conjunction with anti-miR-200b, and found that the downregulation of miR-15b in diabetic mouse wounds induced upregulation of VEGF, VEGFR, angiopoietin-1, and its receptor TEK, and these effects were even more pronounced when anti-miR15b was delivered in conjunction with anti-miR-200b. These results further elucidate the mechanism of miR-15b and suggest a potential synergistic relationship with another anti-angiomiR miR-200b, discussed further below.

#### 4.2.2. miR-20b-5p

In type 2 diabetes, circulating exosomal miR-20b-5p is upregulated and modulates glycogen synthesis and insulin signaling in skeletal muscle *via* AKT and STAT3 signaling pathways, partially characterizing the mechanism of metabolic derangements associated with diabetes (136). Given this association, Xiong et al. (137) explored the role of miR-20b-5p in diabetic wound healing and found that HUVECs treated with exosomal miR-20b-5p isolated from patients with type 2 diabetes demonstrated impaired proliferation and identified wnt9b as a target of miR-20b-5p, which regulates endothelial proliferation, migration, and apoptosis *via* the β-catenin pathway. Diabetic mice treated with these exosomes had significantly slower rates of healing while knockout of miR-20b-5p resulted in improved wound healing (137). Similarly, Liang et al. (138) used exosomes from umbilical cord-derived mesenchymal stem cells (UCMSCs) and enriched them with HIPK3, another transcription factor of VEGF. Treatment of both cell and rodent models with HIPK3-enriched exosomes resulted in decreased apoptosis, improved proliferation, and upregulation of VEGF, and luciferase reporter assays confirmed that HIPK3 bound directly to miR-20b-5p, suggesting that its inhibition ultimately resulted in the demonstrated improvement in angiogenesis (138). Sponging of anti-angiomiRs, as HIPK3 does with miR-20b-5p, demonstrates yet another potential method for developing treatments for diabetic wounds.

#### 4.2.3. miR-23

MiR-23 is highly expressed in endothelial cells and vascular tissue but its role in angiogenesis *in vivo* has been unclear (139). Amin et al. (140) examined the role of the miR-23 family in DFU and found that while miR-23a and miR-23b were downregulated in human tissue biopsies of DFU, miR-23c was upregulated and associated with decreased expression of pro-angiogenic SDF-1α, an association that was confirmed *via* miRNA target prediction. Cai et al. (141) further characterized the potential mechanism of miR-23 in angiogenesis by studying ginsenoside (Rg1), the active ingredient in a plant used in traditional Chinese medicine, through which they found that rats treated with Rg1 exhibited accelerated wound healing. In HUVECs exposed to high glucose, miR-23 was elevated and could also be suppressed with Rg1 treatment, and miR-23 inhibition was associated with increased expression of inducible nitric oxide synthase (iNOS) and VEGF, further suggesting the anti-angiogenic nature of miR-23 (141).

#### 4.2.4. miR-24-3p

Upregulation of miR-24-3p, which can be induced through states of hypoxia, has been associated with impaired angiogenesis in cardiac infarcts and models of limb ischemia by regulating β-catenin signaling and pericyte migration (142). Xu et al. (143) studied the effects of miR-24-3p by isolating exosomes from diabetic patients, in which miR-24-3p was found to be upregulated, and application of the exosomes both to HUVECs and murine cutaneous wounds resulted in impaired cellular migration and tube formation and delayed wound healing, respectively. Additionally, luciferase assays confirmed that miR-24-3p targeted PI3K subunit gamma (PI3KR3), identifying its potential mechanistic involvement in angiogenesis, and ultimately treating wounds in diabetic mice with antagomir-23-3p resulted in improved wound healing (143). Notably, when miR-24-3p inhibition in a murine model of limb ischemia did result in increased vessel formation, however, the new vessels were tortuous and still resulted in impaired perfusion (142). While this was not specifically a diabetic model, one must consider the potential negative effects of anti-angiomiR inhibition when selecting potential therapeutic targets.

#### 4.2.5. miR-26a

MiR-26a has been found to downregulate angiogenesis *via* interactions with VEGF, specifically by reducing VEGF-induced production of nitric oxide (144). In hepatocellular carcinoma (HCC) as well as gastric cancer, miR-26a was found to reduce VEGF production, and miR-26a is also associated with impaired endothelial cell VEGFR2 signaling in HCC (145, 146). MiR-26a has been found to be upregulated in diabetic animal models implicated in multiple complications related to diabetes including angiogenesis (147). Icli et al. (148) identified that exposure of endothelial cells to a high glucose environment resulted in upregulation of miR-26a, which was also upregulated in wounds of diabetic mice, consistent with the impaired angiogenesis known to occur in diabetes. Further, inhibition of miR-26a induced upregulation of angiogenesis and improved wound closure in diabetic mice with consequent increased expression of *SMAD1*, the gene targeted by miR-26a that is associated with upregulated angiogenesis, and decreased expression of the cell cycle inhibitor p27 (148). These findings suggest that downregulation of miR-26a may improve angiogenesis by allowing endothelial proliferation.

#### 4.2.6. miR-92a

MiR-92a, a member of the miR-17/92 cluster discussed previously, has been identified as an anti-angiomiR through *in vitro* studies as well as mouse models of myocardial infarction and limb ischemia, and multiple proangiogenic targets of miR-92a were predicted *via* computational analysis including integrin subunit α5 (ITGA5), which has previously been linked to embryonic angiogenesis (149, 150). MiR-92a has been explored as a potential anti-cancer therapy given its role in suppression of angiogenesis, and delivery of cholesterol-grafted miR-92a to HUVECs resulted in suppression of ITGA5 and protein members of the MAPK pathway, as well as impaired capillary tube formation (151). Gallant-behm et al. (152) utilized inhibitors of miR-92a in the treatment of diabetic wounds in both mouse and pig models and found that wound healing and angiogenesis were significantly improved in animals treated with miR-92a, and that miR-92a inhibition outperformed treatment with recombinant VEGF or PDGF. Lucas et al. (153) utilized a light-activated anti-miR-92a treatment that improved wound healing in a diabetic mouse model through upregulation of wound proliferation and angiogenesis. Interestingly, the effects of the light-activated anti-miR-92a treatment were limited to the wound without changes in miR-92a expression levels seen in other organs, which could pose a potential benefit by preventing unintended miR-92a upregulation that may be associated with pathologic consequence (154). Both studies that explored miR-92a in diabetic models also functioned through the suppression of IGTA5, identifying yet another potential target in the management of diabetic angiogenesis (152, 153).

#### 4.2.7. miR-133b

MiR-133b has been described as an anti-angiomiR in murine models of diabetic retinopathy, in which treatment of retinal microvascular endothelial cells with upregulation of miR-133b resulted in impaired proliferation and migration with upregulation of apoptosis (155). Zhong et al. (156) examined the relationship between miR-133b and EGFR after confirming EGFR as a direct target of miR-133b *via* luciferase assay and observing downregulation of EGFR in high glucose-treated HUVECs. They additionally observed that miR-133b is upregulated in both glucose-treated HUVECs and wounds from human patients with diabetes, and when miR-133b was antagonized in diabetic mice they exhibited significant improvement in wound healing with agomiR-133b having the opposite effect (156). While Zhong et al. (156) describe the other potential mechanisms through which antagonizing miR-133b may induce improved wound healing, this highlights the role of EGFR and its ligand EGF in angiogenesis and wound healing.

#### 4.2.8. miR-135a-3p

MiR-135a has been identified as an inhibitor of angiogenesis in metastatic gastric cancer, specifically though modulation of p53 and focal adhesion kinase (FAK), which activates MAPK and subsequently VEGF (154). In lung cancer, miR-135a is downregulated, resulting in upregulation of the PI3K pathway and increased tumor cell proliferation due to inhibited apoptosis (157). Icli et al. (158) specifically explored miR-135a-3p as an inhibitor of angiogenesis in diabetes and found it to be significantly upregulated in both *in vivo* and *in vitro* studies with impairment of p38 signaling in endothelial cells. Furthermore, inhibition of miR-135a-3p in diabetic mouse wounds resulted in improved wound healing with increased granulation tissue formation and angiogenesis as demonstrated by increased CD31 positivity (158). Icli et al. (158) identified huntingtin-interacting protein 1 (HIP1) as the specific target of miR-135a-3p through which this anti-angiomiR interacts with the p38-MAPK signaling pathway resulting in downregulation of angiogenesis.

#### 4.2.9. miR-139-5p

MiR-139-5p has been implicated as a tumor suppressor in numerous cancers and as a regulator of myogenesis in hypertrophic cardiomyopathy (159, 160). Its anti-angiogenic properties have been previously described in cerebrovascular disease, with overexpression of miR-139-5p resulting in reduced proliferation and angiogenesis of intracranial aneurysm (160). MiR-139-5p has been found to be overexpressed in diabetic models and subsequently identified as a suppressor of angiogenesis (161, 162). Luo et al. (161) demonstrated upregulation of miR-139-5p in multiple diabetic models including endothelial cells derived from human diabetic patients and aortas of diabetic rats, as well as HUVECs cultured in high glucose. When treated with miR-139-5p mimics, cell models demonstrated impaired endothelial proliferation and tube formation with decreased expression of VEGF and PDGF, while treatment with inhibitors of miR-139-5p produced the opposite effect both in cell models and *in vivo* studies of diabetic mice with hind limb ischemia (161). Mechanistically, miR-139-5p was found to target c-jun, a transcription factor involved in the expression of VEGF (161). Liang et al. (162) identified a separate mechanism through which miR-139-5p may enact its anti-angiogenic effects by studying BMSCs derived from diabetic rats, in which miR-139-5p was found to target and downregulate the VEGF regulator HOXA9 and c-fos, a relative of c-jun that similarly affects VEGF and PDGF expression. Furthermore, diabetic rats treated with BMSCs that had been transfected with a miR-139-5p antagomir exhibited improved rates of wound healing as well as increased capillary density and upregulated expression of HOXA9, c-fos, and VEGF, all further solidifying the role of miR-139-5p as an inhibitor of angiogenesis and potential target for therapeutics (162).

#### 4.2.10. miR-195-5p

MiR-195-5p has been extensively studied for its roles in solid tumors and has been found to modulate angiogenesis in breast, gastric, lung, and prostate cancers through numerous proposed mechanisms, including β-catenin pathway suppression and direct targeting of VEGF (163, 164). In breast cancer specifically, extracellular vesicles (EV), which are microparticles present in body fluids including the tumor microenvironment and are capable of acting as a delivery system of various biological molecules, have been implicated in both the pathogenesis of the cancer as well as the effects of chemotherapy (163). EVs have also been tied to the pathogenesis of DFU. Liu et al. (165) isolated EVs from the wound fluid of DFU from human patients to create DF-EVs—HUVECs exposed to DF-EVs exhibited impaired cell migration and decreased vessel formation, while diabetic rats treated with DF-EVs exhibited impaired microvascular formation within granulation tissue. Both miR-195-5p and miR-205-5p were found to be upregulated in DF-EVs, resulting in impaired angiogenesis through direct targeting of VEGFA expression (165).

#### 4.2.11. miR-200

MiR-200 has been linked to impaired angiogenesis in diabetes *via* multiple mechanisms. Chan et al. (166) recognized downregulation of miR-200b in endothelial cells along wound edges and then identified globin transcription factor binding protein 2 (GATA2) and VEGFR2 as its targets, first through computational prediction and then confirmed by luciferase assay and western blot analysis. This pathway was impaired in diabetic wounds with upregulation of miR-200b and silencing of GATA2 and VEGF2R, and these effects were reversed when treated with an anti-miR-200b, creating a wound healing profile more consistent with non-diabetic models (166). Wang et al. (167) describe a separate mechanism through which miR-200 impairs angiogenesis by utilizing bone marrow-derived progenitor cells (BMPCs) from diabetic mice. Diabetic BMPCs were deficient inositol-requiring enzyme 1 (IRE1α), which is responsible for cleaving and thereby inactivating certain mRNAs and miRNAs, including miR-200 along with miR-466, both of which suppress the proangiogenic factor Ang-1 (167). Therefore, therapies that induced upregulation of IRE1α resulted in the degradation of miR-200 and miR-466, which reduced suppression of Ang-1 and ultimately improved angiogenesis and wound healing (167). Finally, as discussed previously, Pizzino et al. (135) identified anti-miR-200b, both alone and in conjunction with anti-miR-15b, resulted in significant upregulation of VEGF and VEGFR2 in addition to Ang-1 and its receptor TEK with overall improvement in wound closure. These studies highlight the multifactorial impact of miR-200 on angiogenesis and diabetic wound healing.

#### 4.2.12. miR-205-5p

The antiangiogenic impact of miR-205 has been described in multiple cancers (168, 169). In thyroid cancer, miR-205 is significantly under-expressed compared to normal thyroid controls, and the under-expression of miR-205 resulted in over-expression of *VEGFA* (168). Transfection of miR-205 into thyroid cancer cells resulted in downregulation of *VEGFA* with downstream inhibition of cell cycle progression and increased apoptosis (168). In gastric cancer, miR-205-5p was also found to be downregulated which was associated with increased CD31 expression (169). When miR-205-5p was upregulated, angiogenesis was impaired *via* suppression of VEGFA and FGF levels by downregulation of ERK signaling (169). As mentioned above, miR-205-5p is upregulated in wound fluids from human DFU and directly target VEGF to inhibit angiogenesis (165). Zhu et al. (170) further explored its antiangiogenic effects by depleting MSCs of miR-205-5p by utilizing an antisense miR-205-5p. By knocking down expression of miR-205-5p, they significantly augmented the therapeutic effect of MSCs in the treatment of wounded diabetic mice with increased expression of VEGF (170).

#### 4.2.13. miR-383

MiR-383 directly targets VEGFA and has been implicated as a regulator of angiogenesis in the highly vascular central nervous system tumor malignant glioma, with overexpression of miR-383 resulting in profound downregulation of proliferation, migration, and tube formation of cellular models of malignant glioma (171). Downregulation of miR-383 also resulted in improved angiogenesis in models of spinal cord injury in rats (172). Han et al. (173) explored the effects of miR-383 in diabetic wound healing by studying its interactions with the long non-coding RNA krüppel-like factor 3 antisense RNA 1 (KLF3-AS1) delivered by BMSC-derived exosomes, and they found that these exosomes promoted increased proliferation, migration, and tube formation in HUVECs grown in high glucose media. Improved angiogenesis and wound healing were noted when diabetic mice were treated with exosomal KLF3-ASI, which notably induced decreased miR-383 expression and upregulation of VEGFA (173). This further illustrates the anti-angiogenic role of miR-383 and solidifies its proposed VEGFA target.

#### 4.2.14. miR-489-3p

While there is limited previous literature describing the relationship between miR-489-3p and angiogenesis, its downregulation was associated with improved angiogenesis and wound healing in a rat model of DFU (174). Huang et al. (174) studied Rg1 as a treatment for DFU, and similarly to its effects on miR-23 as described above, treatment with Rg1 was associated with miR-489-3p downregulation and upregulation of its direct target Sirt1. Therefore, the proposed mechanism by which miR-489-3p is therefore proposed to affect angiogenesis is through inhibition of Sirt1 and subsequent downregulation of PI3K/AKT/eNOS signaling, which is reversed by treatment with Rg1 (174).

#### 4.2.15. miR-615-5p

MiR-615 has been identified as a regulator of embryonic angiogenesis and implicated in both cancer and neural repair (175). MiR-615-5p downregulates multiple pro-angiogenic factors including Tie2, insulin-like growth factor 2 (IGF-2), and *MEF2A* (176). Icli et al. (177) explored the regulation of endothelial cells by miR-615-5p, which is upregulated in the wounds of diabetic mice. Mechanistically, miR-615-5p was found to impair endothelial migration and proliferation by targeting IGF2 as well as ras-associating domain family member 2 (RASSF2), thereby inhibiting VEGF/endothelial nitric oxide synthase (eNOS) signaling (177). Finally, inhibition of miR-615-5p resulted in markedly improved wound closure rates and elevated numbers of CD31+ cells (177).

## 5. Discussion

Chronic diabetic wounds such as DFU affect nearly a third of patients with diabetes and pose an incredible burden to healthcare systems across the globe (2, 9, 10). It is therefore imperative to focus efforts not only on characterizing the mechanism of impaired healing in diabetes but also developing appropriately targeted treatments. As discussed throughout this review, the impairment of angiogenesis is a critical mechanism through which impaired wound healing occurs in diabetes. While recognizing this fact is key to progressing toward the correction of impaired diabetic wound healing, it is clear that there are a vast number of potential factors that contribute to this problem, and it is therefore challenging to narrow down the best mechanism through which impaired angiogenesis may be targeted. Furthermore, the current available treatments for diabetic wounds are minimal and focus mainly on mitigating damage that has already been done, such as maintaining a moist wound environment and debriding devitalized tissue, rather than correcting the pathology at its source (3, 5, 11, 12). EGF has been utilized as the basis for therapeutics in diabetic wounds since 1989, though concerns for potential malignancy have limited its use in the United States (46, 51). Despite this, topical EGF products are available in other parts of the world and have demonstrated benefit, and the injectable Heberprot-P, which was developed and tested in Cuba, has been associated with decreased rates of diabetes-related amputation (51). While becaplermin (recombinant PDGF) has been available as the only FDA-approved pharmaceutical treatment for DFU, its clinical efficacy has been limited and it targets only a single potential mechanism of impaired angiogenesis (13, 92). Over recent years, miRNAs been implicated in the pathology of numerous diseases and have become a source of interest for their treatment potential (15, 18). In this review, we discussed several miRNAs that have been targeted for their roles in aberrant diabetic angiogenesis. One way that this could be accomplished is by directly delivering a pro-angiomiR into the wound bed. Numerous mechanisms have been explored regarding the optimal delivery of pro-angiomiRs. For example, Dewberry et al. (125) utilized the conjugation of miR-146a to a cerium oxide nanoparticle, which not only allows for the stable delivery of miR-146a *via* multiple formulations, including injection and topical application, but also capitalizes on the free radical-scavenging properties of cerium oxide to enhance diabetic wound healing through more than one mechanism. Other methods focus on the sole delivery of the selected miRNA, through either exosomes or microvesicles, which act as membrane-bound drug delivery vehicles (111, 119, 122). Conversely, rather than delivering a pro-angiomiR into the wound bed, treatment methods could instead focus on inhibition of anti-angiomiRs to subsequently upregulate angiogenesis. Lucas et al. (153) pose a particularly interesting option for the delivery of an anti-angiomiR into the wound bed that is light activated, thereby eliminating the inhibition of its target miRNA in other tissues potentially resulting in unintended negative consequences. Additionally, there is the potential to target multiple miRNAs with a single treatment, such as the dual-targeting anti-miR-15b combined with anti-miR-200b employed by Pizzino et al. (135) with improved wound healing in a diabetic mouse model. One could also consider combining multiple miRNAs discussed above, even combining pro-angiomiRs and anti-angiomiRs, to target multiple mechanisms affecting diabetic wound healing at once.

In conclusion, microRNAs play a critical role in the regulation of angiogenesis and can become dysregulated in pathologic conditions such as diabetes mellitus. MicroRNAs may offer a prospective therapeutic target for the treatment of impaired angiogenesis in chronic diabetic wounds, and they allow for the potential to target numerous mechanisms involved in aberrant angiogenesis. While much work remains to be done, the research discussed in this review provides significant promise regarding the treatment of DFU.

## Author contributions

BL, AV, and JB contributed to the conceptualization of the manuscript. BL and JB performed investigation and data curation. BL prepared the original draft of the manuscript. BL, AV, JB, AA, LG, CZ, and KL reviewed and edited the manuscript. CZ and KL performed supervision of the manuscript. All authors contributed to manuscript revision, read, and approved the submitted version.
